# Patterns and Drivers of *nirK*-Type and *nirS*-Type Denitrifier Community Assembly along an Elevation Gradient

**DOI:** 10.1128/mSystems.00667-21

**Published:** 2021-11-02

**Authors:** Yongping Kou, Yanjiao Liu, Jiabao Li, Chaonan Li, Bo Tu, Minjie Yao, Xiangzhen Li

**Affiliations:** a Key Laboratory of Environmental and Applied Microbiology, CAS, Environmental Microbiology Key Laboratory of Sichuan Province, Chengdu Institute of Biology, Chinese Academy of Sciences, Chengdu, China; b Engineering Research Center of Soil Remediation of Fujian Province University, College of Resources and Environment, Fujian Agriculture and Forestry University, Fuzhou, China; California Department of Water Resources

**Keywords:** *nirK*-type denitrifiers, *nirS*-type denitrifiers, community assembly, elevation gradient, denitrification potential

## Abstract

*nirK*-type and *nirS*-type denitrifier communities mediate the conversion of nitrite to nitric oxide, which is the key step in denitrification. Results of previous studies have indicated that *nirK*-type and *nirS*-type denitrifiers may occupy different niches; however, the mechanisms and drivers of their responses to environmental changes within community assembly are poorly understood. In this study, we evaluated the distribution and assembly of *nirK*-type and *nirS*-type denitrifier communities along an elevation gradient from 1,800 to 4,100 m at Mount Gongga, China. Results showed that elevational patterns of alpha diversity in *nirK*-type and *nirS*-type denitrifier communities followed hump-backed patterns along the elevation gradient. However, *nirK*-type denitrifier communities formed two distinct clusters that were primarily separated by elevation, whereas *nirS*-type denitrifier communities formed three distinct clusters that were primarily separated by forest type along the elevation gradient. Moreover, deterministic processes were dominant in governing the assemblages of *nirK*-type and *nirS*-type denitrifiers. Soil pH was a key factor influencing the alpha and beta diversity of the *nirK*-type denitrifier communities, whereas plant richness was a primary variable influencing *nirS*-type denitrifiers. Additionally, our work revealed that soil denitrification potential was mainly explained by the variation in the beta diversity of denitrifier communities rather than the alpha diversity of denitrifier communities or denitrifier abundances over a large elevation gradient, and *nirK*-type denitrifiers played more important roles in soil denitrification. These results may contribute to predicting the consequences of global changes on denitrifier communities and their ecological services.

**IMPORTANCE** Mount Gongga is the highest peak in the Hengduan Mountain region and is located at the southeastern fringe of the Tibetan Plateau, Sichuan Province, southwest China. As a transitional zone between the Tibetan Plateau and Sichuan Basin, Gongga Mountain features particularly diverse topography, geology, climate, and biodiversity and is a globally significant hot spot of biodiversity. In this contribution, we comprehensively describe the diversity and assembly of denitrifier communities along an elevation gradient on Gongga Mountain. Our findings established for the first time that the distribution patterns of beta diversity and driving factors differed between *nirK*-type and *nirS*-type denitrifier communities, and deterministic processes were dominant in shaping communities of denitrifiers. Moreover, the beta diversity of denitrifier communities rather than alpha diversity or denitrifier abundance played an important role in explaining denitrification potential, and the beta diversity of *nirK*-type denitrifier communities was more important than *nirS*-type denitrifier communities in soil denitrification. This work provides crucial insights into the spatial distribution of denitrifier communities and their ecological function and increases our understanding of the mechanisms underlying spatial distribution of community assembly along large elevation gradients.

## INTRODUCTION

Soil denitrifiers are crucial in the reduction of nitrate (NO_3_^−^) and/or nitrite (NO_2_^−^) to gaseous nitrogen (N_2_) during denitrification (1). The reduction of NO_2_^−^ to nitric oxide (NO), the key step in denitrification, is catalyzed by nitrite reductase encoded by the *nirK* or *nirS* gene (2). Therefore, *nirK* and *nirS* are typically used as effective marker genes for characterizing the abundances and community composition of denitrifiers in ecosystems (3, 4). These two genes are considered mutually exclusive among denitrifiers (5), and most denitrifiers possess either *nirK* or *nirS*, although a few strains have been reported to possess both genes (6). In addition, these two genes are thought to occur in two ecologically distinct denitrifying groups, and denitrifiers with *nirK* and *nirS* genes may differ in different denitrification abilities (7–9). However, the linkages among biogeographic distributions, assembly processes, and key drivers of soil denitrifier communities as well as the potential denitrification rate (PDR) in ecosystems remain unclear.

Previous studies have shown that *nirK*-type and *nirS*-type denitrifier communities in estuary, watershed, and agricultural ecosystems respond differently to environmental variables, such as soil type, climate, organic carbon, nitrate, plants, oxygen concentration, and salinity (7, 10–12). For example, Azziz et al. (10) reported that *nirS*-type denitrifier communities are more sensitive than the *nirK*-type denitrifiers to soil type, rice cultivar, and water management. Moreover, *nirS*-type denitrifiers are more likely to be influenced by plant species than *nirK*-type denitrifiers in terrestrial ecosystems (11, 13–15). Plant communities influence soil microbial assemblages either through the effects of rhizodeposits or the alteration of soil conditions (11, 16). Indeed, Hou et al. (11) showed the *nirS*-type denitrifiers to be more sensitive than *nirK*-type denitrifiers to the rhizosphere effect in agricultural soils and proposed that root exudates, acting as inducible carbon sources, can exhibit different effects on *nirS*-type and *nirK*-type denitrifier communities. In contrast, a previous study reported a stronger effect on *nirK*-type denitrifiers than *nirS*-type denitrifiers in the rhizosphere of a wetland plant (17). Similarly, soil pH also shows different effects on the abundances and communities of *nirK*-type and *nirS*-type denitrifiers in various ecosystems (4, 18, 19). For example, *nirS*-type denitrifier communities are more sensitive to pH gradients (ranging from pH 4.2 to 6.6) than are *nirK*-type denitrifiers under a long-term (50 years) pH manipulation (4). In addition, Ligi et al. (20) reported that the abundance of the *nirS* gene, but not that of the *nirK* gene, was affected by soil pH in a constructed riverine wetland complex. These inconsistent results may be attributed to different selection mechanisms for various denitrifiers in specific ecosystems.

Community assembly processes include deterministic and stochastic processes (21, 22). According to the framework described in previous studies (21), deterministic processes include heterogeneous and homogenous selection, whereas stochastic processes include dispersal limitation, homogenizing dispersal, and drift. Homogeneous selection (under homogeneous conditions) results in lower variation in community structure or species/compositional turnover, whereas heterogeneous selection under heterogeneous environmental conditions produces high variation in community structure. Mountain ecosystems exhibit great changes in climate, plant parameters, and soil properties over short spatial distances, which can be used as analogs to environmental gradients to understand microbial activity, community assembly, and their relationships with environmental factors. Previous studies have revealed that deterministic processes (heterogeneous selection) dominate in the assembly processes of soil microbial communities (including communities of soil bacteria, diazotrophs, and methanotrophs) along a large altitudinal gradient, among which climate, plant, and soil parameters play important roles in shaping microbial communities (23–25). Moreover, along with environmental factors (such as temperature and plant parameters) that covary with elevation and could influence the distribution patterns of soil microbial communities (23, 26), geological processes (such as parent rock and weathering) explain additional variation in plant and microbial communities (27). However, the relative contributions of deterministic versus stochastic processes in the assemblies of *nirK*-type and *nirS*-type denitrifier communities along large elevation gradients remain unknown.

Various elevational biodiversity (species richness) patterns of soil bacterial communities (23, 28–30) and functional microbial groups, e.g., diazotrophs (25) and methanotrophs (24), have been observed, suggesting that microbial diversity patterns along elevation gradients are ecosystem specific and scale dependent. However, previous studies have primarily focused on the changes in elevational biodiversity patterns in terms of species richness (*α*-diversity), and the elevational changes in microbial community turnover (*β*-diversity) have been much less studied (31). Moreover, some studies have suggested that the *β*-diversity of denitrifier communities is a more robust indicator for interpreting the variation in PDR than denitrifier abundances in arid and semiarid regions (32, 33). However, denitrifier gene (*nirK* or *nirS*) abundances, but not denitrifier communities, are reported to be good predictors of PDR (1, 34). For example, studies have reported PDR to be correlated with the abundance of *nirS*-type denitrifiers or *nirK*-*nirS* gene abundance in fertilized grassland soil (35) and in soils from a permafrost black spruce forest to a rich fen (1), whereas another study also found PDR to be positively correlated with the abundance of *nirK*-type denitrifiers but not correlated with changes in corresponding community composition in forest soils (34). In addition, previous studies have suggested that *nirS*-type denitrifiers are more likely to be capable of complete denitrification under suitable conditions than are *nirK*-type denitrifiers (36). Therefore, *nirS*-type denitrifiers may play more important roles in denitrification, since they can produce greater quantities of denitrification enzyme than can the *nirK* community (4). However, the relative contributions of *nirK*-type and *nirS*-type denitrifier communities/abundances to PDR remain poorly understood along elevation gradients.

Mount Gongga is the highest mountain on the eastern boundary of the Tibetan Plateau. The drastic environmental changes along the elevation gradient on the eastern slope of Mount Gongga offer a unique platform for investigating the biogeographical distributions of *nirK*-type and *nirS*-type denitrifiers and the ecological processes regulating community assembly over such a large elevational scale. Hence, our primary objectives were to compare the biogeographical distributions, assembly processes, and key driving factors of the variations of *nirK*-type and *nirS*-type denitrifiers and to assess the relationships between variation in *nirK*-type and *nirS*-type denitrifiers and PDR along the elevation gradient. Specifically, we hypothesized (i) that deterministic processes (heterogeneous selection) dominate the assembly processes of denitrifier communities and that soil pH and plant parameters are the key environmental factors shaping *nirK*-type and *nirS*-type denitrifier communities along an elevation gradient, and (ii) that PDR is mainly explained by variation in the beta diversity of denitrifier communities rather than by denitrifier abundances or the alpha diversity of denitrifier communities along a large elevation gradient and that *nirS*-type denitrifiers play more important roles in soil denitrification.

## RESULTS

### Climate, plant, and soil properties along the elevation gradient.

The mean annual temperature (MAT) decreased and the mean annual precipitation (MAP) increased with elevated altitude. The average total carbon (TC), total nitrogen (TN), extractable nitrate ion (NO_3_^−^), and conductivity were significantly higher at low elevations (1,800 to 2,800 m) than those at high elevations (3,000 to 4,100 m). Soil pH (ranging from 3.53 to 7.23) was significantly higher at 1,800 to 2,600 m than at 2,800 to 4,100 m. The extractable ammonium ion (NH_4_^+^) levels varied from 1.81 to 62.78 mg (kg dry weight soil)^−1^ along the elevation gradient. Among the woody plant species, the evergreen broadleaf trees (EB), the deciduous broadleaf trees (DB), and dark coniferous trees (DC) exhibited an uneven distribution at all elevations. Plant richness first decreased and then increased with increasing altitude. These data were from Li et al. (23).

### The *α*-diversity, community composition, and abundances of *nirK*-type and *nirS*-type denitrifiers.

**FIG 1:**
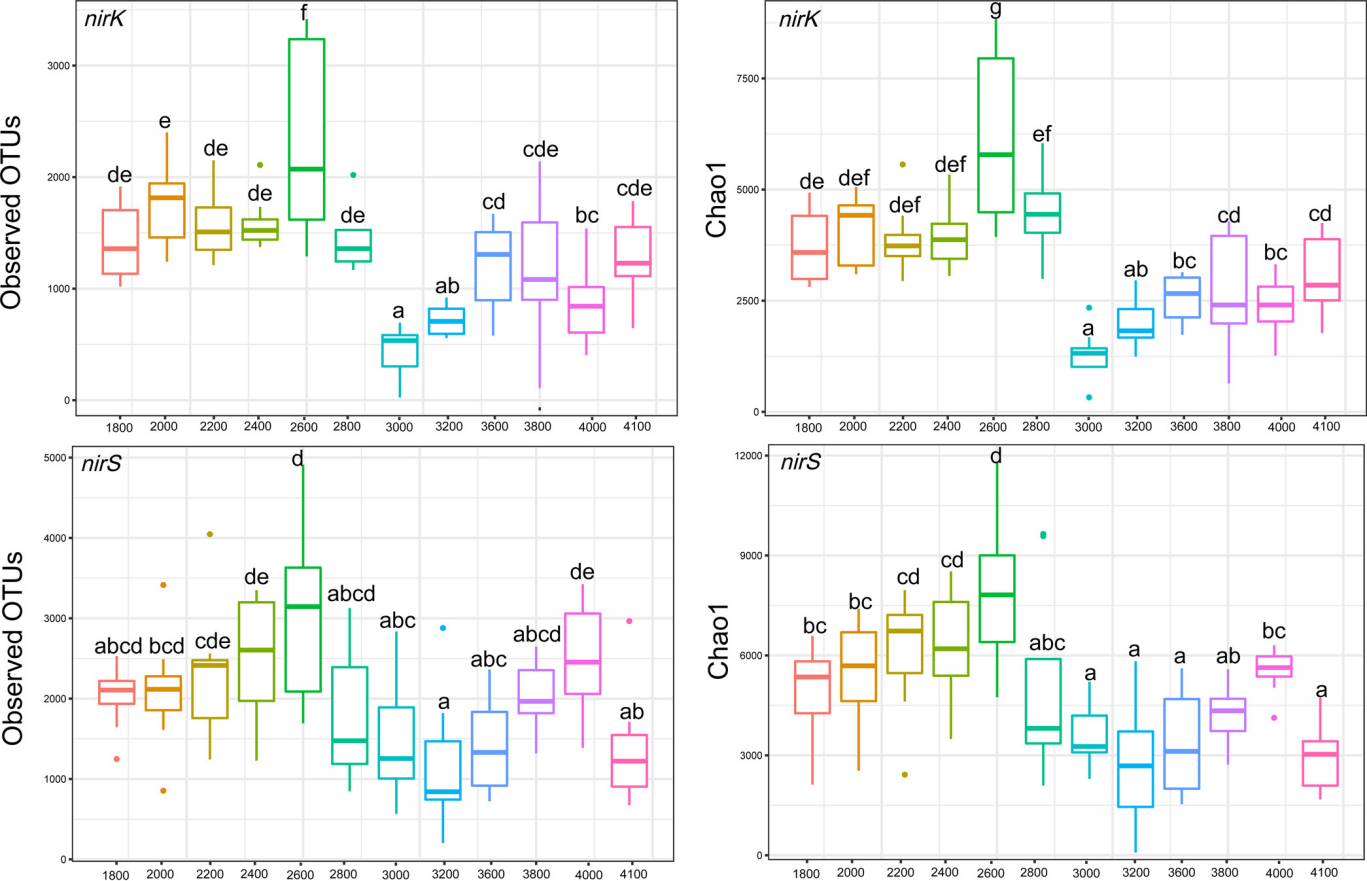
*α*-Diversity indices for *nirK*-type and *nirS*-type denitrifier communities, including observed operational taxonomic units (OTUs) and Chao1 along the elevation gradient.

**FIG 2:**
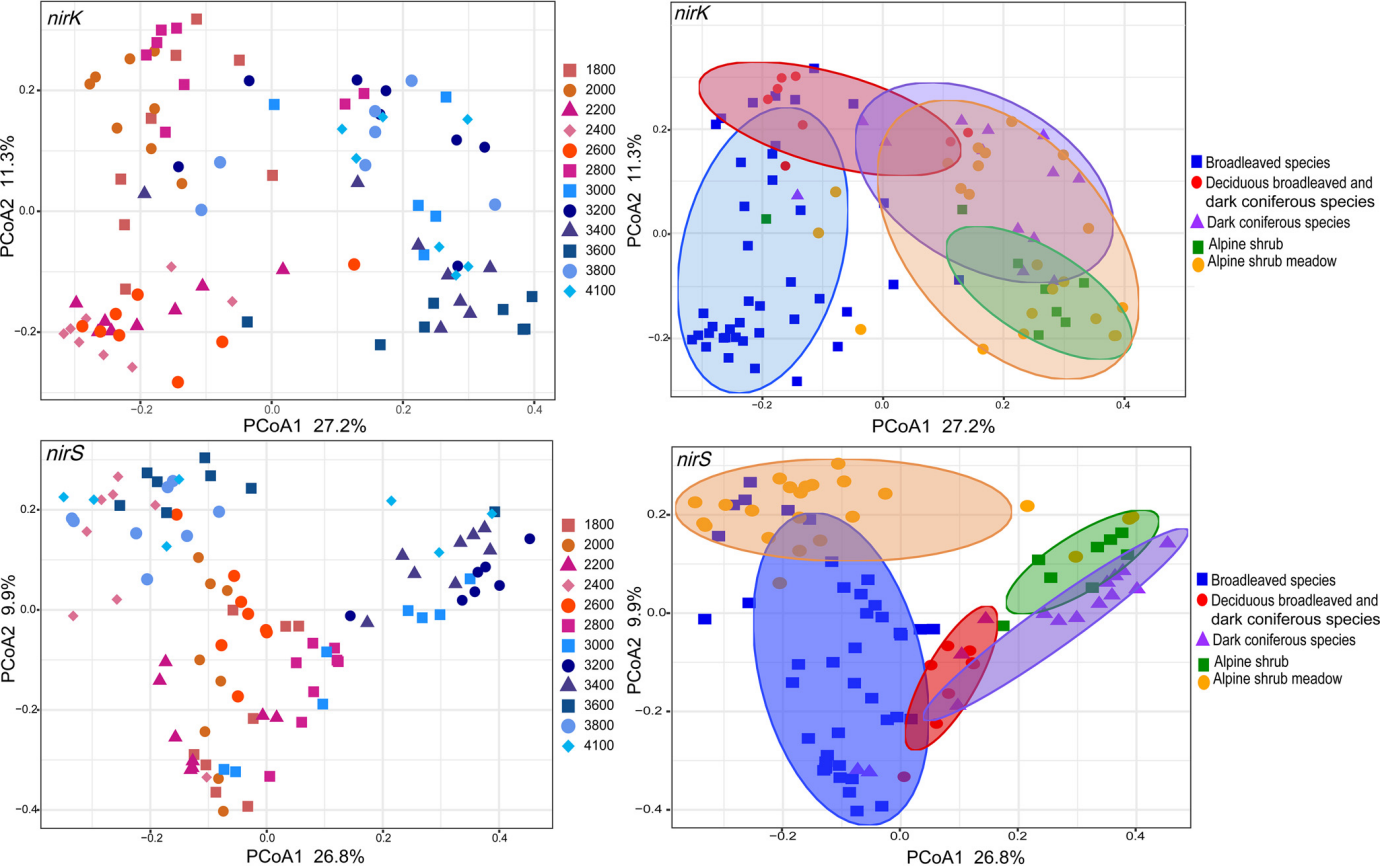
Principal coordinate analysis (PCoA) of *nirK*-type and *nirS*-type denitrifier community composition along the elevation gradient, ranked by elevation (upper and lower right) and vegetation type (upper and lower left) based on the Bray-Curtis dissimilarity matrix. Ellipses were defined as 95% confidence intervals for the centroids of denitrifiers.

The *α*-diversities of *nirK*-type and *nirS*-type denitrifier communities exhibited hump-backed patterns along the elevation gradient, and the peak values occurred at 2,600 and 3,800 m, respectively (Fig. 1). Moreover, an abrupt decrease in alpha diversity of denitrifier communities occurred between 2,600 and 2,800 m. However, the results of nonparametric multivariate analysis of variance (NPMANOVA) showed that the *nirK*-type and *nirS*-type denitrifier communities changed significantly with elevation, except for those in several neighboring sites (Fig. 2; see also Tables S1 and S2 in the supplemental material). The *nirK*-type denitrifier communities formed two distinct clusters that were primarily separated by elevation, and they were also separated by the presence of mixed forests of deciduous broadleaf and dark coniferous species at 2,800 m (Fig. 2). However, *nirS*-type denitrifier communities formed three distinct clusters that were separated primarily by deciduous broadleaf/dark coniferous forests and alpine shrub meadows along the elevation gradient (Fig. 2).

10.1128/mSystems.00667-21.5 TABLE S1 A nonparametric multivariate analysis of variance to test the variation in the *nirK*-type community structure (*β*-diversity) among 12 different elevations. Download Table S1, DOCX file, 0.03 MB. Copyright © 2021 Kou et al. 2021 Kou et al. https://creativecommons.org/licenses/by/4.0/ This content is distributed under the terms of the Creative Commons Attribution 4.0 International license.

**FIG 3:**
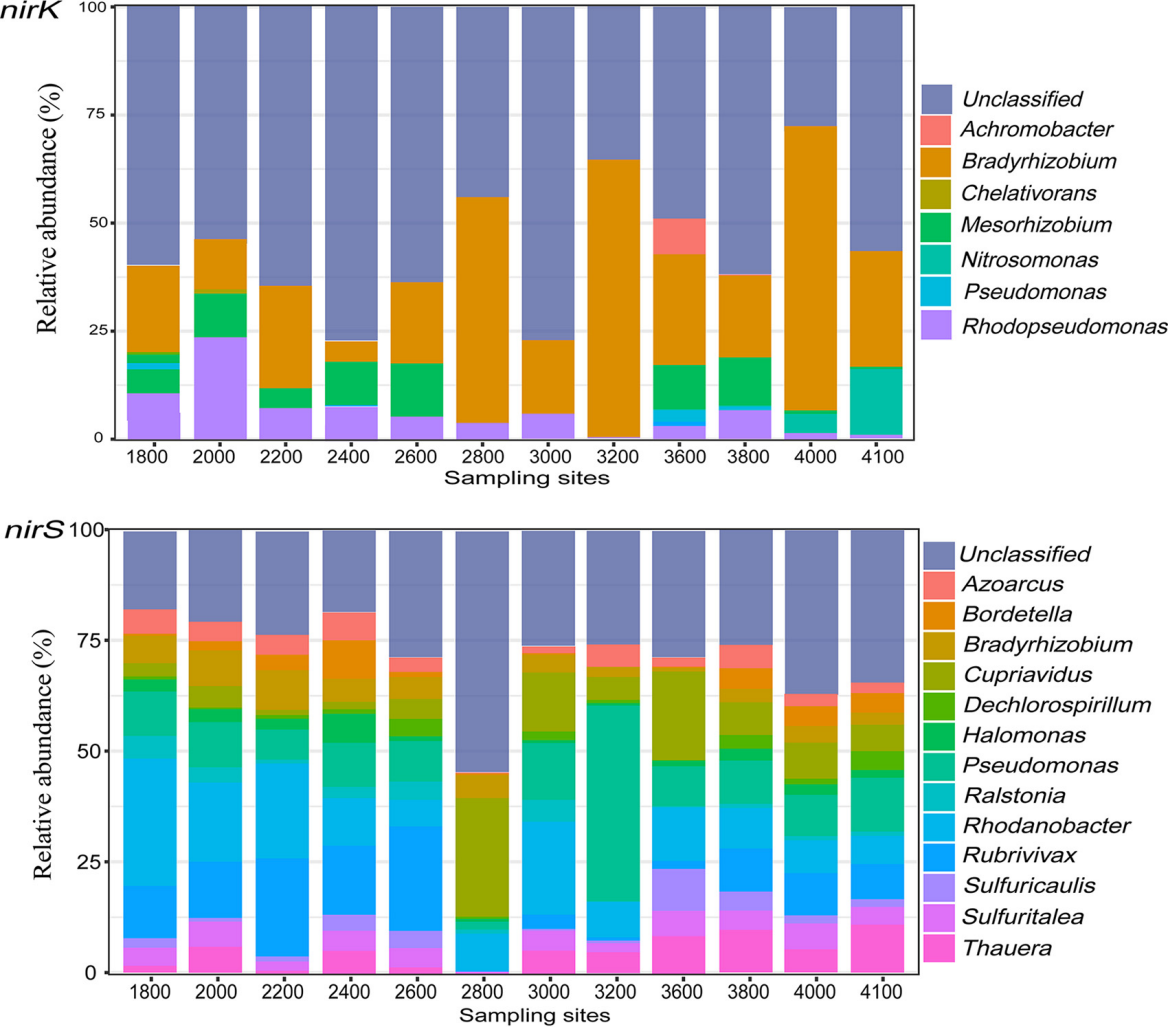
Community composition of *nirK*-type and *nirS*-type denitrifiers at the genus level along an elevation gradient.

The *nirK*-type denitrifier communities were dominated by class *Alphaproteobacteria* (relative abundance, 63 to 88%) (Fig. S1), followed by *Betaproteobacteria* (3 to 10%) and *Gammaproteobacteria* (0.1 to 5%) (Fig. S1). In this study, the genera with relative abundance of ≥0.05% at least at one elevation were defined as major genera. The major genera of *nirK*-type denitrifiers were *Achromobacter*, *Bradyrhizobium*, *Chelativorans*, *Mesorhizobium*, *Nitrosomonas*, Pseudomonas, and *Rhodopseudomonas* (Fig. 3). The *nirS*-type denitrifier communities were dominated by classes *Betaproteobacteria* (23 to 82%) and *Gammaproteobacteria* (9 to 68%) (Fig. S1), whereas *Alphaproteobacteria* only accounted for 2 to 13% (Fig. S1). The major genera of *nirS*-type denitrifiers included *Azoarcus*, *Bordetella*, *Bradyrhizobium*, *Cupriavidus*, *Dechlorospirillum*, *Halomonas*, Pseudomonas, *Ralstonia*, *Rhodanobacter*, *Rubrivivax*, *Sulfuricaulis*, *Sulfuritalea*, and *Thauera* (Fig. 3).

10.1128/mSystems.00667-21.1 FIG S1 Community composition of *nirK*-type and *nirS*-type denitrifiers at the class level along the elevation gradient. The relative abundance of each class was the average of all replicate samples in each elevation gradient. Download FIG S1, TIF file, 0.8 MB. Copyright © 2021 Kou et al. 2021 Kou et al. https://creativecommons.org/licenses/by/4.0/ This content is distributed under the terms of the Creative Commons Attribution 4.0 International license.

The different patterns of *nirK*-type and *nirS*-type denitrifiers along the elevation gradient were also reflected in specific taxa (Fig. S2 and S3). Among the major genera, only *Bradyrhizobium* and Pseudomonas occurred in both *nirK*-type and *nirS*-type denitrifier communities (Fig. 3). However, *nirK*-type denitrifiers in *Bradyrhizobium* and Pseudomonas showed response patterns to elevation differing from those of *nirS*-type denitrifiers. Specifically, the relative abundance of *nirK*-type *Bradyrhizobium* increased significantly with elevation, whereas the relative abundance of *nirS*-type *Bradyrhizobium* decreased significantly with elevation. The presence of *nirK*-type Pseudomonas was only detected at high elevations of 3,600 and 3,800 m, with relative abundance first increasing and then decreasing, whereas the relative abundance of *nirS*-type Pseudomonas increased at a low elevation and then decreased at elevations above 3,200 m (Fig. S2 and S3).

10.1128/mSystems.00667-21.2 FIG S2 Variations in the relative abundances of main genera belonging to *nirK*-type denitrifiers along the elevation gradient. Lines represent best-fit regressions of relative abundances in each sample at each elevation site versus elevations. Download FIG S2, TIF file, 1.0 MB. Copyright © 2021 Kou et al. 2021 Kou et al. https://creativecommons.org/licenses/by/4.0/ This content is distributed under the terms of the Creative Commons Attribution 4.0 International license.

10.1128/mSystems.00667-21.3 FIG S3 Variations in the relative abundances of main genera belonging to *nirS*-type denitrifiers along the elevation gradient. Lines represent best-fit regressions of relative abundances in each sample at each elevation site versus elevations. Download FIG S3, TIF file, 2.4 MB. Copyright © 2021 Kou et al. 2021 Kou et al. https://creativecommons.org/licenses/by/4.0/ This content is distributed under the terms of the Creative Commons Attribution 4.0 International license.

The numbers of copies of *nirK* and *nirS* genes decreased with increasing altitude, ranging from (1.40 ± 0.46) × 10^8^ to (8.89 ± 4.89) × 10^8^ copies g^−1 ^dry soil and (1.05 ± 0.26) × 10^7^ to (1.05 ± 6.61) × 10^7^ copies g^−1 ^dry soil, respectively (Table S3). The abundances of the *nirK* genes were 16.67 ± 9.82 to 44.99 ± 11.77 times greater than those of *nirS* genes at all elevations (Table S3).

10.1128/mSystems.00667-21.6 TABLE S2 A nonparametric multivariate analysis of variance to test the variation in the *nirS*-type community structure (*β*-diversity) among 12 different elevations. Download Table S2, DOCX file, 0.03 MB. Copyright © 2021 Kou et al. 2021 Kou et al. https://creativecommons.org/licenses/by/4.0/ This content is distributed under the terms of the Creative Commons Attribution 4.0 International license.

10.1128/mSystems.00667-21.7 TABLE S3 Copy numbers of *nirK* (10^8^ copies g^−1^ dry soil) and *nirS* genes (10^7^ copies g^−1^ dry soil) and the ratio of *nirK* to *nirS* genes in soil samples. Download Table S3, DOCX file, 0.03 MB. Copyright © 2021 Kou et al. 2021 Kou et al. https://creativecommons.org/licenses/by/4.0/ This content is distributed under the terms of the Creative Commons Attribution 4.0 International license.

### Ecological processes shaping denitrifier community assemblies.

**FIG 4:**
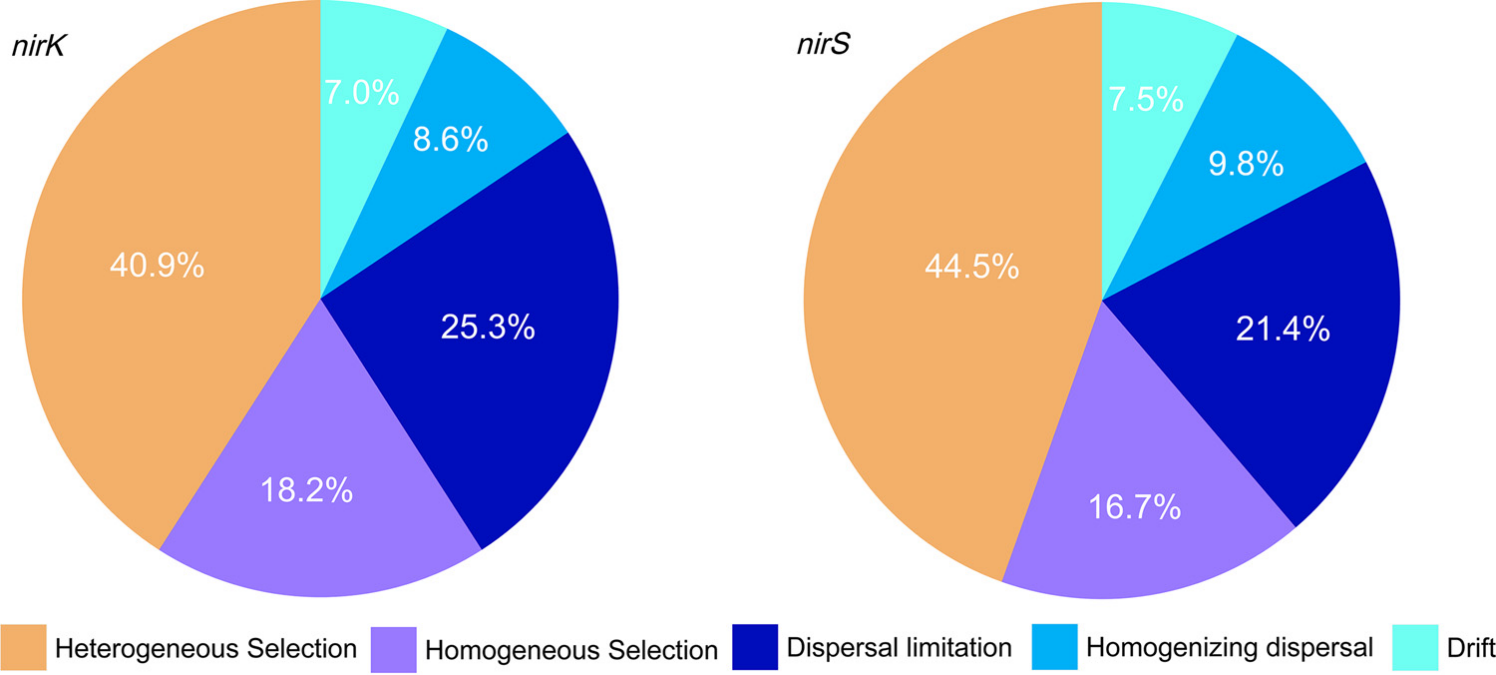
Phylogenetic null model approach was used to quantify the relative contributions of deterministic processes (including heterogeneous selection and homogeneous selection) and stochastic processes (including dispersal limitation, homogenizing dispersal, and drift) to *nirK*-type and *nirS*-type denitrifier community assembly.

For *nirK*-type and *nirS*-type denitrifier communities, the mean nearest taxon index (NTI) was 1.27 or 1.38, respectively (*P *< 0.05), indicating that both *nirK*-type and *nirS*-type communities were phylogenetically clustered and that the assemblies of *nirK*-type and *nirS*-type denitrifier communities were affected mainly by environmental filtration. Moreover, based on the results of phylogenetic null model analysis, heterogeneous selection and homogeneous selection explained 40.9% and 18.2%, respectively, of the turnover in community composition for *nirK*-type denitrifiers, with 25.3%, 8.6%, and 7% explained by dispersal limitation, homogenizing dispersal, and undominated, respectively (Fig. 4). For the *nirS*-type denitrifier communities, heterogeneous and homogeneous selection explained 44.5% and 16.7% of the community turnover, followed by 21.4%, 9.8%, and 7.5% explained by dispersal limitation, homogenizing dispersal, and undominated, respectively (Fig. 4).

### Factors influencing the community composition and abundances of *nirK*-type and *nirS*-type denitrifiers along the elevation gradient.

Most observed environmental factors were significantly associated with community-level attributes and the abundances of denitrifiers (Table S4). Among all the variables, soil pH and the total diameter at breast height (DBH) in DC (DBH-DC) forest explained the most variation in the *α*-diversity of *nirK*-type denitrifier communities, whereas plant richness was the main predictor of the *α*-diversity of *nirS*-type denitrifiers (Table 1). In addition, the DBH in DB (DBH-DB) and TC/TN explained the most variation in number of copies of *nirK* (Table 1), whereas TN and plant richness were the major predictors for the *nirS* (Table 1). Soil pH showed a significant correlation with the relative abundances of *Bradyrhizobium*, *Chelativorans*, and *Rhodopseudomonas* in the *nirK*-type communities (Table S5), whereas plant richness was significantly correlated with the relative abundances of *Bradyrhizobium*, *Rubrivivax*, *Cupriavidus*, *Ralstonia*, *Halomonas*, and *Thauera* in the *nirS*-type communities (Table S6).

**TABLE 1:** Roles of environmental variables on *α*-diversity and copy numbers of the *nirK* and *nirS* genes evaluated by stepwise multivariate regression modeling

Gene	*α*-Diversity/copy no. test	Explanatory variable	Contribution of the individual predictor^*a*^ (%)	*P* value	Adjusted *R*^2^ for full model (*P* value)
*nirK*	Chao 1	pH	13.1	0.001	0.20 (<0.0001)
		DBH-DC	7.2	0.050	
	Observed OTUS	pH	10.6	0.012	0.20 (<0.0001)
		DBH-DC	9.5	0.024	
	Copy no.	DBH-DB	19.2	0.028	0.35 (<0.0001)
		TC/TN	16.0	0.000	
*nirS*	Chao 1	Plant richness	30.1	0.000	0.40 (<0.0001)
		NO_3_^−^-N	9.9	0.002	
	Observed OTUS	Plant richness	15.9	0.000	0.23 (<0.0001)
		NO_3_^−^-N	6.8	0.007	
		MAT	0.5	0.046	
	Copy no.	TN	21.3	0.001	0.51 (<0.0001)
		Plant richness	17.4	0.008	
		Cond	7.5	0.008	
		DBH-EB	4.5	0.017	

a Percentage of the total sum of squares explained by each variable. DBH represents the total diameter at breast height, while DBH-DB, DBH-EB, and DBH-DC represent the percentage representation of deciduous broadleaf trees, evergreen broadleaf trees, and dark coniferous trees, respectively, in total DBH. TC, total carbon; TN, total nitrogen; MAT, mean annual temperature; Cond, Conductivity.

10.1128/mSystems.00667-21.8 TABLE S4 Spearman rank correlation analysis showing the relationships between *α*-diversity and the gene copy numbers and environmental attributes. Download Table S4, DOCX file, 0.03 MB. Copyright © 2021 Kou et al. 2021 Kou et al. https://creativecommons.org/licenses/by/4.0/ This content is distributed under the terms of the Creative Commons Attribution 4.0 International license.

10.1128/mSystems.00667-21.9 TABLE S5 Spearman rank correlation analysis showing the relationships between the relative abundances of *nirK*-type denitrifier taxa at the genus level and environmental factors along the elevation gradient. Download Table S5, DOCX file, 0.03 MB. Copyright © 2021 Kou et al. 2021 Kou et al. https://creativecommons.org/licenses/by/4.0/ This content is distributed under the terms of the Creative Commons Attribution 4.0 International license.

10.1128/mSystems.00667-21.10 TABLE S6 Spearman rank correlation analysis showing the relationships between the relative abundances of *nirS*-type denitrifier taxa at the genus level and environmental factors along the elevation gradient. Download Table S6, DOCX file, 0.03 MB. Copyright © 2021 Kou et al. 2021 Kou et al. https://creativecommons.org/licenses/by/4.0/ This content is distributed under the terms of the Creative Commons Attribution 4.0 International license.

**FIG 5:**
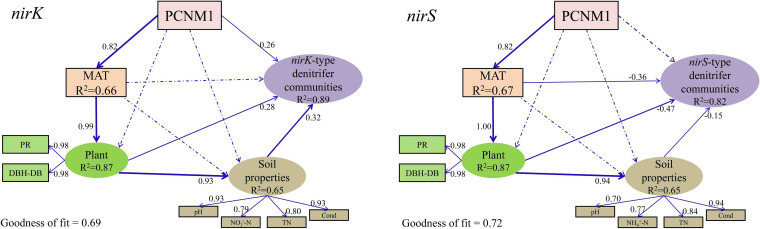
Partial least-squares path modeling (PLS_PM) was used to estimate the direct and indirect effects of climatic, plant, and soil properties on *nirK*-type and *nirS*-type denitrifier communities. The width of an arrow represents the strength of the path coefficient. Continuous and dashed arrows indicate significant and nonsignificant path coefficients, respectively. PCNM1, principal component of neighbor matrices, representing geographical distance; MAT, mean annual temperature; PR, plant richness; TN, total nitrogen; Cond, conductivity. DBH represents the total diameter at breast height. DB represents the percentage of total DBH accounted for by deciduous broadleaf trees.

**FIG 6:**
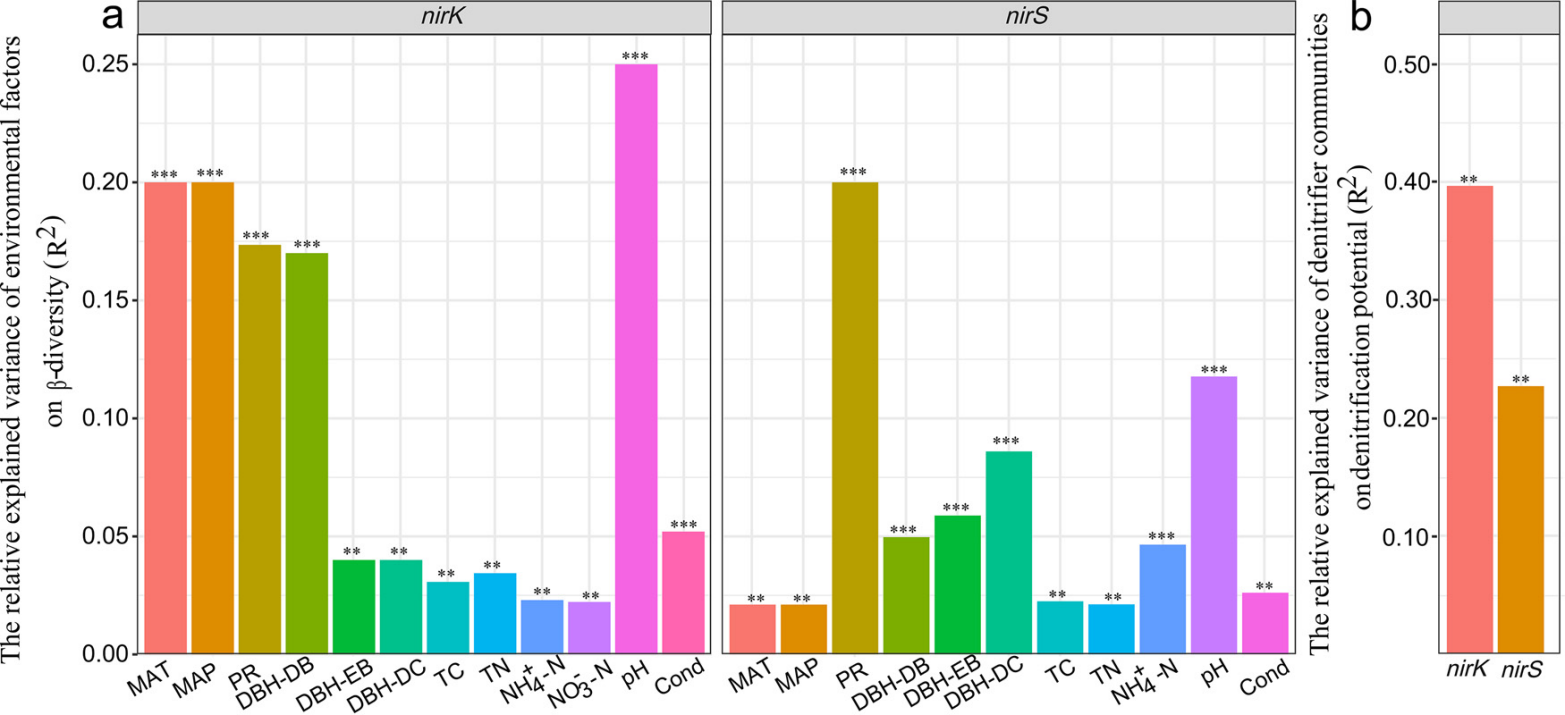
Multiple regression on distance matrices (MRM) was used to estimate the relative proportions of variance explained by environmental factors in the turnover of *nirK*-type and *nirS*-type denitrifier communities along the altitudinal gradient (a) and the relative variation in nitrification potential explained by *nirK*-type and *nirS*-type denitrifier communities (b) in the low- and high-altitudinal sections, respectively. MAT, mean annual temperature; MAP, mean annual precipitation; PR, plant richness; TC, total carbon; TN, total nitrogen; Cond, conductivity. DBH represents the total diameter at breast height. DB, EB, and DC represent the percent representations of deciduous broadleaf trees, evergreen broadleaf trees, and dark coniferous trees, respectively, in total DBH. **, *P *< 0.01; ***, *P *< 0.001.

Partial least-squares path modeling (PLS_PM) explained 89% and 82% of the variation in *nirK*-type and *nirS*-type denitrifier communities, respectively (Fig. 5) (goodness of fit = 0.69 and 0.72, respectively). Geographical distance (PCNM1) significantly influenced *nirK*-type denitrifiers directly or indirectly through its effect on climate (MAT), MAT significantly influenced *nirK*-type denitrifiers indirectly through its effect on plants, and plants significantly influenced *nirK*-type denitrifier communities directly or indirectly through their effects on soil properties (Fig. 5). The direct effect of soil properties (path coefficient of 0.32) on the *nirK*-type denitrifiers exceeded those of PCNM1 (path coefficient of 0.26) and plants (path coefficient of 0.28) (Fig. 5). Among the environmental variables, soil pH was the most important factor shaping *nirK*-type communities (Fig. 6, Table 2).

**TABLE 2:** Partial Mantel test analysis between the *nirK*- and *nirS*-type denitrifier communities and environmental factors along the elevation gradient^*a*^

Factor	*nirK*-type communities		*nirS*-type communities	
	pMantel.r	*P* value	pMantel.r	*P* value
Elevation	0.02	0.22	−0.07	1.00
Longitude	0.11	0.01*	0.24	0.00**
Latitude	0.07	0.04*	0.11	0.01*
pH	0.28	0.00**	0.29	0.00**
NH_4_^+^-N	0.10	0.02*	0.16	0.00**
NO_3_^−^-N	−0.04	0.76	0.10	0.04*
TC	0.03	0.23	0.12	0.01*
TN	0.03	0.20	0.11	0.01*
TC/TN	0.05	0.14	0.04	0.21
Conductivity	0.05	0.14	0.14	0.01*
MAT	−0.08	1.00	−0.10	1.00
MAP	0.02	0.21	−0.05	0.99
Plant richness	0.15	0.00**	0.36	0.00**
DBH-DB	0.03	0.23	0.19	0.00**
DBH-EB	0.03	0.26	0.18	0.00**
DBH-DC	0.13	0.00**	0.31	0.00**

a TC, total carbon; TN, total nitrogen; MAT, mean annual air temperature; MAP, mean annual precipitation. DBH represents the total diameter at breast height, while DBH-DB, DBH-EB, and DBH-DC represent the percent representation of deciduous broadleaf trees, evergreen broadleaf trees, and dark coniferous trees, respectively, in total DBH. *, *P*< 0.05; **, *P* < 0.01.

Geographical distance (PCNM1) significantly influenced the *nirS*-type communities indirectly by its effect on climate (MAT). MAT significantly influenced *nirS*-type denitrifiers directly or indirectly by its effect on plants, and plants significantly influenced *nirS*-type denitrifier communities directly or indirectly by their effects on soil properties (Fig. 5). The direct effect of plants (path coefficient of −0.47) on *nirS*-type denitrifiers was greater than those of MAT (path coefficient of −0.36) and soil properties (path coefficient of −0.15) (Fig. 5). Among the environmental variables, plant richness was the main factor shaping *nirS*-type denitrifier communities (Fig. 6, Table 2).

The results of cooccurrence network analysis showed that positive correlations dominated in relationships between *nirK*-type and *nirS*-type denitrifiers (Fig. S4). Moreover, plant richness played an important role in influencing the cooccurrence relationships between *nirK*-type and *nirS*-type denitrifiers (Fig. S4).

10.1128/mSystems.00667-21.4 FIG S4 Cooccurrence network analysis between *nirK*-type and *nirS*-type denitrifiers. Each node represents an operational taxonomic unit (OTU), which signifies a species. Each line connects two OTUs. The red and green lines represent negative and positive correlations, respectively. TC, total carbon; TN, total nitrogen; MAT, mean annual temperature; MAP, mean annual precipitation. DBH-DB, DBH-EB, and DBH-DC represent the percent representation of deciduous broadleaf trees, evergreen broadleaf trees and dark coniferous trees, respectively, in total DBH. DBH represents the total diameter at breast height. Download FIG S4, TIF file, 2.8 MB. Copyright © 2021 Kou et al. 2021 Kou et al. https://creativecommons.org/licenses/by/4.0/ This content is distributed under the terms of the Creative Commons Attribution 4.0 International license.

### Soil denitrification potential and its relationship with denitrifier communities and abundances.

**FIG 7:**
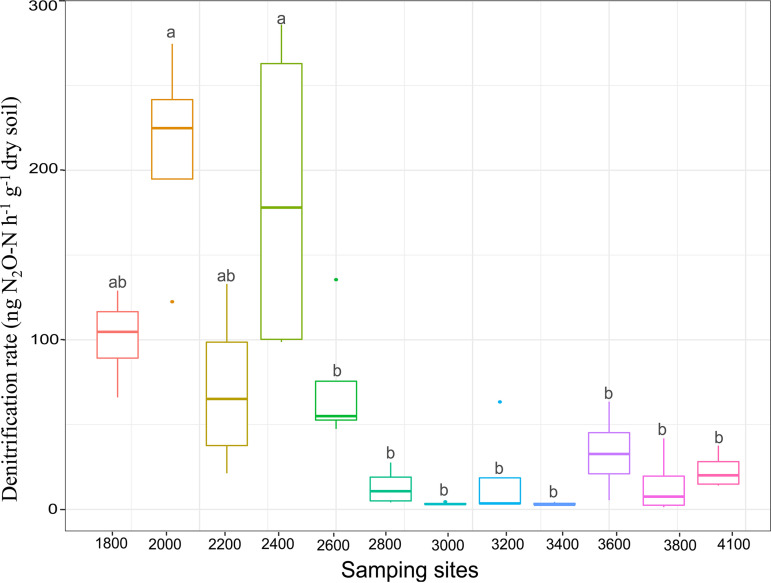
Soil denitrification potential along the elevation gradient.

Based on the acetylene inhibition technique, the PDR was estimated. The results showed that the PDR was significantly higher at low elevations (ranging from 21.30 to 285.96 μg N_2_O-N g^−1 ^dry soil h^−1^, 1,800 to 2,600 m) than at high elevations (ranging from 1.32 to 63.50 μg N_2_O-N g^−1 ^dry soil h^−1^, 2,800 to 4,100 m) (Fig. 7). Results of multiple regression on distance matrices (MRM) showed that the *β*-diversity of *nirK*-type and *nirS*-type denitrifier communities explained PDR well, and *β*-diversity of *nirK*-type denitrifier communities were more important in explaining PDR than were *nirS*-type denitrifiers (Fig. 6). However, the abundances and *α*-diversity of *nirK*-type and *nirS*-type denitrifier communities did not show significant effects on PDR.

## DISCUSSION

### The patterns of assembly of *nirK*-type and *nirS*-type denitrifier communities along the elevation gradient.

The *α*-diversity of the *nirK*-type and *nirS*-type denitrifier communities showed hump-backed patterns along the elevation gradient. Moreover, an abrupt decrease in the *α*-diversity of denitrifier communities occurred between 2,600 and 2,800 m (Fig. 1). Similar patterns have also been reported for the elevational changes of soil bacteria (28), diazotrophs (25), and methanotrophs (24) along the same elevation gradient. Such a pattern of variation may be ascribed to the significant differences in soil properties (such as pH, NO_3_^−^-N, TC, TN, and conductivity) between low (1,800 to 2,800 m) and high (3,000 to 4,100 m) elevations (23). Indeed, the variations in these soil properties significantly influenced *nirK*-type and *nirS*-type denitrifiers (Fig. 6; see also Tables S4 to S6 in the supplemental material), consistent with the findings of previous studies (7, 10–12). In addition, plants can influence *nirK*-type and *nirS*-type denitrifier communities (11, 14, 15). In this study, plants changed from mixed forests of deciduous broadleaf and coniferous species to uniformly coniferous forests at 2,850 m, suggesting that the changes in plant community at approximately 2,850 m contribute to the differentiation in *α*-diversity of denitrifier communities between low and high elevations. In addition to the contemporary factors described above, the elevational breakpoint of denitrifier diversity at approximately 2,800 m coincided with the Indus-Yalu suture zone fault in this region, which implies that past geological processes (e.g., parent rock and weathering) have left a strong signature on the elevational patterns of soil microbial diversity (27).

Moreover, *nirK*-type denitrifier communities formed two clusters at low (1,800 to 2,800 m) and high (3,000 to 4,100 m) elevational sections (Fig. 2). Similar results have been reported for the distribution of soil bacterial and methanotrophic communities along the same elevation gradients (23, 24), and differences in community structure between the lower and higher elevations were attributed mainly to changes in soil pH. However, unlike the *nirK*-type denitrifiers, three clusters of *nirS*-type denitrifier communities were observed along the elevation gradient (Fig. 2), which was consistent with the changes in plant communities. The differences in elevation pattern between the *nirS*-type and *nirK*-type denitrifier communities suggest that they are adapted to different ecological niches. *nirK*-type denitrifier communities were dominated by class *Alphaproteobacteria*, whereas the *nirS*-type denitrifier communities were dominated by classes *Betaproteobacteria* and *Gammaproteobacteria* (Fig. S1). This is consistent with the report by Heylen et al. (37). The major genera *Bradyrhizobium* and Pseudomonas showed different patterns of response to elevation between *nirK*-type and *nirS*-type denitrifiers (Fig. S2 and S3). The relative abundance of *nirK*-type *Bradyrhizobium* was primarily correlated with soil pH, whereas relative abundances of *nirS*-type *Bradyrhizobium* were primarily correlated with plant richness (Table S6). These results further support the possibility that they are adapted to different ecological niches.

### Deterministic processes dominating the assemblies of *nirK*-type and *nirS*-type denitrifier communities.

This study proved that *nirK*-type and *nirS*-type denitrifier communities were phylogenetically clustered. Deterministic processes (heterogeneous selection) were dominant in shaping *nirK*-type and *nirS*-type communities over the elevation gradient, and similar results have also been observed for soil bacteria (23, 30), diazotrophs (25), and methanotrophs (24) along the same elevation gradient. Furthermore, heterogeneous selection explained a high fraction of turnover in community composition of *nirK*-type and *nirS*-type denitrifiers (Fig. 4). These results provide partial support for hypothesis i. Natural selection was not able to explain denitrifier community structure completely because the drastic variation in climate, vegetation, and soil properties over a short spatial distance along the elevation gradient resulted in high variation in community structure. In addition, microbes may have ubiquitous dispersal capabilities due to their size and lower degree of restriction by geographical barriers (38); however, microbes have been recognized to be dispersal limited through a modeling approach (39). Indeed, this study demonstrated that dispersal limitation was also important in explaining the variations in the *nirK*-type and *nirS*-type denitrifier communities in montane ecosystems (Fig. 4). Therefore, our results confirmed the interactive effect of heterogeneous selection and dispersal limitation in shaping soil microbial communities.

### Different key drivers shifting *nirK*-type and *nirS*-type denitrifier communities.

Soil pH was the primary environmental variable explaining the variation in *nirK*-type denitrifier communities (Fig. 6, Tables 1 and 2). Indeed, previous studies have demonstrated the importance of pH in shaping denitrifier communities and other microbial groups in various ecosystems (4, 40, 41). Appropriate pH is crucial for the growth and activity of denitrifiers (4), and it is considered the primary variable that links soil organic matter recycling, plant nutrition, and plant-microbial interactions in soils (13, 42). For example, soil pH can influence the level of dissolved organic matter by affecting the sorption of dissolved organic matter components to soil molecules (13). Changes in nutrient availability can influence the abundances and community composition of denitrifiers (4). However, there is a debate about the relative effects of pH and climate (temperature) on soil microbial communities (43). In this study, PLS_PM analysis showed that the direct effect of a soil property (pH) on *nirK*-type denitrifier communities was greater than that of climate (Fig. 5). Moreover, soil pH showed a significant correlation with the relative abundances of *Bradyrhizobium*, *Chelativorans*, and *Rhodopseudomonas* (Table S5). These *nirK*-type denitrifiers were abundant generalists found in all the samples (Fig. S2). Therefore, soil pH may directly mediate *nirK*-type denitrifiers by species sorting mechanisms that shift the relative abundances of *Bradyrhizobium*, *Chelativorans*, and *Rhodopseudomonas*.

Plant richness was the main factor explaining the variation in the *nirS*-type denitrifier communities (Fig. 6, Tables 1 and 2). The results described above provide support for hypothesis i. The pattern of distribution of *nirS*-type denitrifiers was consistent with spatial variation in the plant community, which suggests that *nirS*-type denitrifiers are more sensitive to plant properties than are *nirK*-type denitrifiers in mountain ecosystems. Previous studies have reported *nirS*-type denitrifiers to be more closely associated than *nirK*-type denitrifiers with plants in forest soils (13), watershed soils (14), agricultural soils (11), and wetland soils (15), although some other studies have shown that *nirS*-type denitrifiers are sensitive to chemical and physical properties (44, 45). Genomic data have shown that the *nirS*-type denitrifiers may have a more complete denitrification pathway than that of *nirK*-type denitrifiers (46), and the majority of *nirS*-type denitrifiers are anaerobic heterotrophic microorganisms that can grow on the exudation of labile carbon (10, 11). Plants influence denitrifier communities not only by directly increasing the amount and quality of plant aboveground (litter) and belowground (root exudation) materials (11, 16, 47) but also by indirectly modifying soil physicochemical properties, including soil permeability, pH, substrate availability, and soil moisture (48–50). Plant richness was significantly correlated with the relative abundances of *Bradyrhizobium*, *Rubrivivax*, *Cupriavidus*, *Ralstonia*, *Halomonas*, and *Thauera* (Table S6), which were abundant *nirS*-type denitrifiers widely distributed along the elevation gradient (Fig. S3). Therefore, plants may directly influence *nirS*-type denitrifiers by shifting the abundances of these microbes and indirectly by their effects on soil properties. Additionally, plant richness also played an important role in influencing the cooccurrence of *nirK*-type and *nirS*-type denitrifiers along elevation gradients (Fig. S4), suggesting that the cooccurrence relationships between *nirK*-type and *nirS*-type denitrifiers will readily suffer the effects of variation in plant communities in mountain ecosystems in the future.

### Relationships between denitrifier communities and soil denitrification potential.

In this study, the distinct changes in PDR were significantly associated with the changes in *β*-diversity of *nirK-*type and *nirS*-type denitrifier communities (Fig. 6). This provides partial support for hypothesis ii. This result was consistent with those of recent studies that have found *nirK*-type and *nirS*-type communities to play important roles in determining denitrification rates in grassland systems (35) and in pasture soils (32). Our results suggest that the predictive strength of models explaining facultative processes could be improved by taking into account denitrifier communities. Moreover, the *β*-diversity of the *nirK*-type denitrifiers explained more of the variation in PDR than did that of *nirS*-type denitrifiers, implicating more important roles of *nirK*-type denitrifiers in soil denitrification. This result did not support hypothesis ii, that the composition of *nirS*-type communities plays more important roles in explaining PDR. This result was also inconsistent with finding of previous studies that have reported that *nirS*-type denitrifiers were able to produce greater quantities of denitrification enzyme and, thus, maintain higher PDR (4) and that *nirS*-type denitrifiers are more likely than *nirK*-type denitrifiers to be capable of complete denitrification (36). These inconsistent results may be ascribed to the increased sensitivity to pH of transcription found in *nirS*-type denitrifiers, with transcription limited at low pH (for example, pH 4.7) (4, 51); therefore, *nirS*-type denitrifiers have poor capacity to reduce NO_2_^−^ to NO at low pH values. Unlike *nirS*-type denitrifiers, *nirK*-type denitrifiers did not show sensitivity to soil pH; therefore, *nirK*-type denitrifiers may have a competitive advantage at low pH values. However, the abundances of denitrifiers did not show significant correlations with PDR. This finding is inconsistent with previous reports that gene abundance can be used as an integrative ecological variable to predict the dynamics of PDR (1, 7, 33). This contradiction might be ascribed to the dominant effects of environmental variables on PDR, since the fluctuations of certain environmental factors (such as soil pH and total organic carbon) may result in simultaneous changes in the denitrification rate but not denitrifier gene abundances (33).

### Conclusions.

This study revealed elevation patterns of *nirK*-type and *nirS*-type denitrifier communities along the elevation gradient on Mount Gongga. We have found, for the first time, that deterministic processes, mainly heterogeneous selection, were more important than other processes in shaping the assemblies of *nirK*-type and *nirS*-type denitrifier communities. Moreover, the primary influencing variables were pH for *nirK*-type denitrifiers and plant richness for *nirS*-type denitrifiers. In addition, the *β*-diversity of *nirK*-type denitrifier communities explained more variation in PDR than that of *nirS*-type denitrifier communities. These results indicate close linkages among denitrifier diversity, climate, plant richness, and soil properties, which are critical for predicting the consequences of global changes on denitrifier communities and their ecological functions.

## MATERIALS AND METHODS

### Site description, sample collection, and soil characterization.

Mount Gongga (29°33′ to 29°36′ N, 101°57′ to 102°05′ E) is located on the eastern boundary of the Tibetan Plateau, Sichuan Province, southwest China. Mount Gongga is also the easternmost 7,556-m peak in the world and the third highest peak outside the Himalayan/Karakoram Range, after Tirich Mir and Kongur Tagh. The eastern slope of Mount Gongga is relatively steep (average slope, 75%), and the western slope is less steep (average slope, 25%). The mean annual temperature on the eastern slope of Mount Gongga decreases by 0.67°C when the elevation increases by 100 m, whereas the mean annual precipitation increases by 67.5 mm. Climatic and topographic variation create a vertical zonation of different forest types, with the vegetation on the east aspect of Mount Gongga representing the complete vegetation spectrum of the subtropical region in China. Evergreen broadleaf forests range from 1,200 to 1,800 m and mainly include *Lindera* spp., *Cinnamomum* spp., *Cyclobalanopsis* spp., etc. Mixed evergreen and deciduous broadleaf forests range from 1,800 to 2,500 m and mainly include *Lithocarpus cleistocarpus* and *Quercus* spp. Mixed forests of deciduous broadleaf and coniferous species range from 2,500 to 2,850 m and mainly include *Tsuga dumosa*, *Picea brachytyla*, and *Acer flabellatum*. From 2,850 m up to the treeline at approximately 3,850 m, the species *Abies fabri* is dominant in the subalpine forests. From 3,600 to 3,700 m, alpine shrubs (*Rhododendron lapponicum*) dominate in the lower region, and mixed mosaics of alpine shrubs and meadows range from 3,650 to 4,200 m (52).

Soil samples were collected in October 2014 from 12 sites along a 1,800- to 4,100-m elevation gradient with a pairwise interval of approximately 200 m along the east slope of Mount Gongga, as described by Li et al. (23). Briefly, at each sampling site, eight 10-m by 10-m plots were established. At each plot, five random soil core samples (0 to 10 cm) were collected using a soil corer (2.5-cm diameter) and then pooled as one composite sample for further analysis. Overall, 96 topsoil samples were collected from 12 sites along the elevation gradient. After passing through a 2-mm sieve, each fresh soil sample was separated into two parts, one of which was stored at 4°C for measuring soil physiochemical properties, whereas the other was stored at −40°C for molecular analysis.

The following climatic, plant, and soil properties were collected or determined and used in subsequent statistical analyses: latitude; longitude; elevation; MAT; MAP; TC, TN, NH_4_^+^, and NO_3_^−^ concentrations; pH; and conductivity. Moreover, the plant species composition and richness were recorded in each plot (23). The diameter at breast height was measured for each woody plant, and the percentages of total DBH of EB, DB, and DC were calculated at each elevation based on the sum of diameters of all the woody plants at each elevation (23). Descriptions of climate data collection and plant and soil property measurements are available in Li et al. (23).

### DNA extraction and qPCR amplification.

Total soil DNA was extracted from 0.25 g soil using a MoBio Powersoil DNA isolation kit (San Diego, CA, USA) by following the manufacturer’s instructions. The concentration and purity of the extracted DNA were quantified using a NanoDrop spectrophotometer and 1% agarose gels, and high-quality DNA was stored at −20°C for downstream analysis. The *nirK* and *nirS* genes were amplified using the primer pairs F1aCu/R3Cu (53) and cd3aF/R3cd (54), respectively. Quantitative PCR (qPCR) is an effective method and is widely used to determine the abundances of denitrifier genes (*nirK* and *nirS*) (1, 55). Despite its high variability, qPCR still allows for a comparative analysis of the relative abundance of each gene across the different soil samples (56). The reaction volume was 10 μl and contained 0.5 μl of each primer, 5 μl of 2× SYBR green qPCR master mix (Bio-Rad, USA), 2 μl of DNA template, and 2 μl of sterilized water. PCR was performed in a thermocycler for 5 min at 95°C, followed by 40 cycles of denaturation at 95°C for 30 s, annealing for 30 s (57 and 55°C for the *nirK* and *nirS* genes, respectively), and extension at 72°C for 30 s. Melting curve analysis was conducted after amplification. The qPCR standards for quantification were obtained from PCR amplification products of genes from environmental DNA using each primer set, and the detailed method is available in Kou et al. (57). Amplification efficiencies were 99% and 98% for the *nirK* and *nirS* genes, respectively, with *R*^2^ values higher than 0.99 and no detection of signals in the negative controls.

### PCR amplification and MiSeq sequencing.

Amplification of *nirK* and *nirS* genes was performed using the gene primers F1aCu/R3Cu (53) and cd3aF/R3cd (54), respectively. The 25-μl reaction system contained 0.3 μM forward and reverse primers, 12.5 μl of 2× EasyTaq PCR SuperMix (TransGen Biotech, China), 25 ng DNA template, and sterile water. Thermal cycling included an initial denaturation at 95°C for 5 min, followed by 35 cycles of amplification (94°C for 30 s, 57°C for *nirK* gene or 55°C for *nirS* gene for 1 min, and 72°C for 3 min) and a final extension at 72°C for 8 min. Four replicate PCR products from the same DNA sample were pooled and purified using a DNA gel extraction kit (Axygen, USA) by following the manufacturer's instructions, and the concentration and purity of DNA were determined using a NanoDrop spectrophotometer. PCR products were mixed in equal amounts and sequenced with an Illumina MiSeq PE300 sequencer by following the 2 × 300 bp paired-end sequencing protocol at Chengdu Institute of Biology, CAS, China. The error rate of the sequencing platform was 1.5% for *nirK* and 2.1% for *nirS*.

### Processing of sequence data.

The QIIME pipeline was used to analyze raw sequences according to the barcodes, with trimming and quality filtering (58). Reads containing any ambiguous bases or any nucleotide mismatches within the barcodes or primer sequences were removed prior to analysis. Reads longer than 300 nucleotides and with high average quality score (Q ≥ 30) were used for further analysis. Chimeric sequences (averages of 2.9 and 4.7% for the *nirK* and *nirS* genes, respectively) were removed using Usearch 8 (59). Nonchimeric sequences with frameshifts (averages of 7.3% for *nirK* and 8.3% for *nirS*) were discarded (60). The analysis described above resulted in 217,082 (*nirK*) and 280,487 (*nirS*) high-quality sequences. All samples were resampled to an equal depth of 1,500 sequences per sample. Operational taxonomic unit (OTU) clustering was performed at a 3% dissimilarity cutoff value based on the nucleotide sequences using the UCHIME algorithm (v4.2.40) (61). Furthermore, the databases for both the nucleotide sequence alignment and species assignments were extracted from NCBI (http://www.ncbi.nlm.nih.gov/) and the Ribosomal Database Project function gene pipeline (http://fungene.cme.msu.edu/) (11, 62). To reduce sequence redundancy in diversity computation, identical *nirK* and *nirS* sequences were dereplicated using PRINSEQ (63). Classification of OTUs was performed using BLAST and the lowest common ancestor (LCA) algorithm in MEGAN (64). Related scripts about the bioinformatic analysis of *nirK* and *nirS* genes are available at http://egcloud.cib.cn and http://lxzgroup.cib.cas.cn/kytj/yjff/.

### Estimation of ecological processes shaping community assembly.

A phylogenetic null model approach was used to quantify the ecological processes shaping community assembly (21, 65, 66). We calculated the nearest taxon index (NTI) of each sample and *β*-nearest taxon index (*β*NTI) for paired samples using the R functions “comdistnt” and “ses.mntd” in the package “picante” (67, 68). The NTI can be used to examine the average taxonomic distance between each species and its closest relative in the tree (69). In general, NTI values significantly greater than zero indicate phylogenetic clustering; conversely, NTI values significantly less than zero indicate greater influence of stochastic processes (21). If *β*NTI > 2 or *β*NTI < −2, deterministic processes are the most important factors in community assembly (21), whereas if |*β*NTI| < 2, stochastic processes are critical in shaping community composition. Specifically, if *β*NTI > 2, pairwise comparisons were evaluated as the contribution of heterogeneous selection, whereas if *β*NTI < −2, pairwise comparisons were estimated as the contribution of homogeneous selection (21). The Raup-Crick metric incorporating the relative abundances of species (RC_bray_) was used to further quantify the stochastic processes (21). If |*β*NTI| < 2 and RC_bray_ > 0.95, pairwise comparisons were quantified as the fraction of the dispersal limitation, whereas if |*β*NTI| < 2 and RC_bray_ < −0.95, pairwise comparisons were quantified as homogenizing dispersal (21, 70). Finally, the fraction of the pairwise comparisons with |*β*NTI| < 2 and |RC_bray_| < 0.95 was treated as undominated (70). The detailed script for the calculation process of ecological processes shaping community assembly can be found on GitHub (https://github.com/ChiLiubio/microeco).

### Potential denitrification rate.

The PDR was determined using the acetylene inhibition technique (71). One hundred grams of fresh soil from each sample was weighed into a 1-liter glass bottle, and the soil moisture was adjusted to 60% of field capacity. Bottles were preincubated with loosely capped stoppers at 25°C for 1 week, and then soil equivalent to 20 g dry soil from each sample was transferred to a separate 250-ml serum bottle. A 5-ml solution containing 1,200 μg ml^−1^ glucose-C and 200 μg ml^−1^ NO_3_^−^-N was added to each bottle (9, 72). All of the serum bottles were sealed and made anoxic by filling with pure N_2_ gas (99.999%) for 2 min. Approximately 10% of the headspace of each bottle was replaced with acetylene to block the conversion of N_2_O to N_2_ during denitrification. At the same time, the gas tightness of the incubation system was determined using a control bottle without soil. At 2 and 4 h, 10 ml headspace gas was taken from each bottle using a syringe. The N_2_O concentrations were measured using a gas chromatograph (GC; Shimadzu, Kyoto, Japan) equipped with an electron capture detector. The PDR values were calculated according to the change in the N_2_O concentration between the 2- and 4-h measurements (71).

### Statistical analyses.

The data were transformed by Box-Cox transformation and subjected to analysis of variance (ANOVA), and Tukey’s *post hoc* test was performed to determine significant (*P *< 0.05) effects. One-way analysis of variance was performed to estimate significant differences (*P *< 0.05) in numbers of copies of *nirK* and *nirS* genes as well as in PDR among 12 different elevation gradients. Spearman’s rank correlation analysis was performed to correlate environmental factors with *α*-diversity indices, the relative abundances of the denitrifier taxa, denitrifier gene abundances, and PDR. The *P* values from the correlation analysis were adjusted according to the Benjamini-Hochberg false discovery rate (FDR) (73). Furthermore, stepwise multivariate regression modeling was used to identify the main factors influencing the *α*-diversity and numbers of copies of the *nirK* and *nirS* genes. Nonlinear fitting was performed between the relative abundances of major genera of *nirK*-type and *nirS*-type denitrifiers and elevation. The detailed scripts for the calculation process of nonliner fitting are available at http://lxzgroup.cib.cas.cn/kytj/yjff/. Principal coordinate analysis (PCoA) based on Bray-Curtis distances was applied to explore the variation in denitrifier communities (*β*-diversity) along the elevation gradient. The statistical significance of differences among 12 elevations was assessed by NPMANOVA, with Bonferroni correction of *P* values for multiple comparisons, in PAST version 2.17.

We performed PLS_PM (74) to evaluate the fit of the *nirK*-type/*nirS*-type denitrifier communities to geographical distance and measured environmental parameters. Principal components of neighbor matrices (PCNM) represent the geographical distance and were calculated using the function “pcnm” in the R package “vegan” (75). The models were constructed using the function “inner plot” in the R package “plspm” (74). The method was described in detail by Kou et al. (57). In addition, MRM was performed to identify the main factors shaping the denitrifier communities at the OTU level using the “MRM” function in the R-library “ecodist.” Furthermore, the effects of variation in *nirK*-type/*nirS*-type denitrifier communities and numbers of copies of *nirK*-type/*nirS*-type denitrifiers on PDR were estimated by MRM. In the MRM model, Euclidean distance matrices and Bray-Curtis distance matrices were used for environmental factors and denitrifier communities, respectively. Partial Mantel test analysis between the *nirK*-type and *nirS*-type denitrifier communities and environmental factors was also performed to further identify the main factors shaping the denitrifier communities along the elevation gradient. In addition, the patterns of cooccurrence between *nirK*-type and *nirS*-type denitrifiers plus environmental factors were determined to further estimate the main factors shaping the denitrifier communities in the molecular ecological network analyses pipeline (MENAP) (http://ieg2.ou.edu/MENA/main.cgi) with random matrix theory (RMT)-based algorithms at the OTU level (76). Cytoscape 3.7.0 software was used to visualize the network graphs.

### Data availability.

The raw sequence data were stored in the European Nucleotide Archive under the accession number PRJEB30869 (http://www.ebi.ac.uk/ena/data/view/PRJEB30869).
