# Supplementary figures and images for: Patterns and Drivers of nirK-Type and nirS-Type Denitrifier Community Assembly along an Elevation Gradient
Source: mSystems. 2021 Nov 2;6(6):e00667-21. doi: 10.1128/mSystems.00667-21 (PMC8562487; doi:10.1128/mSystems.00667-21)

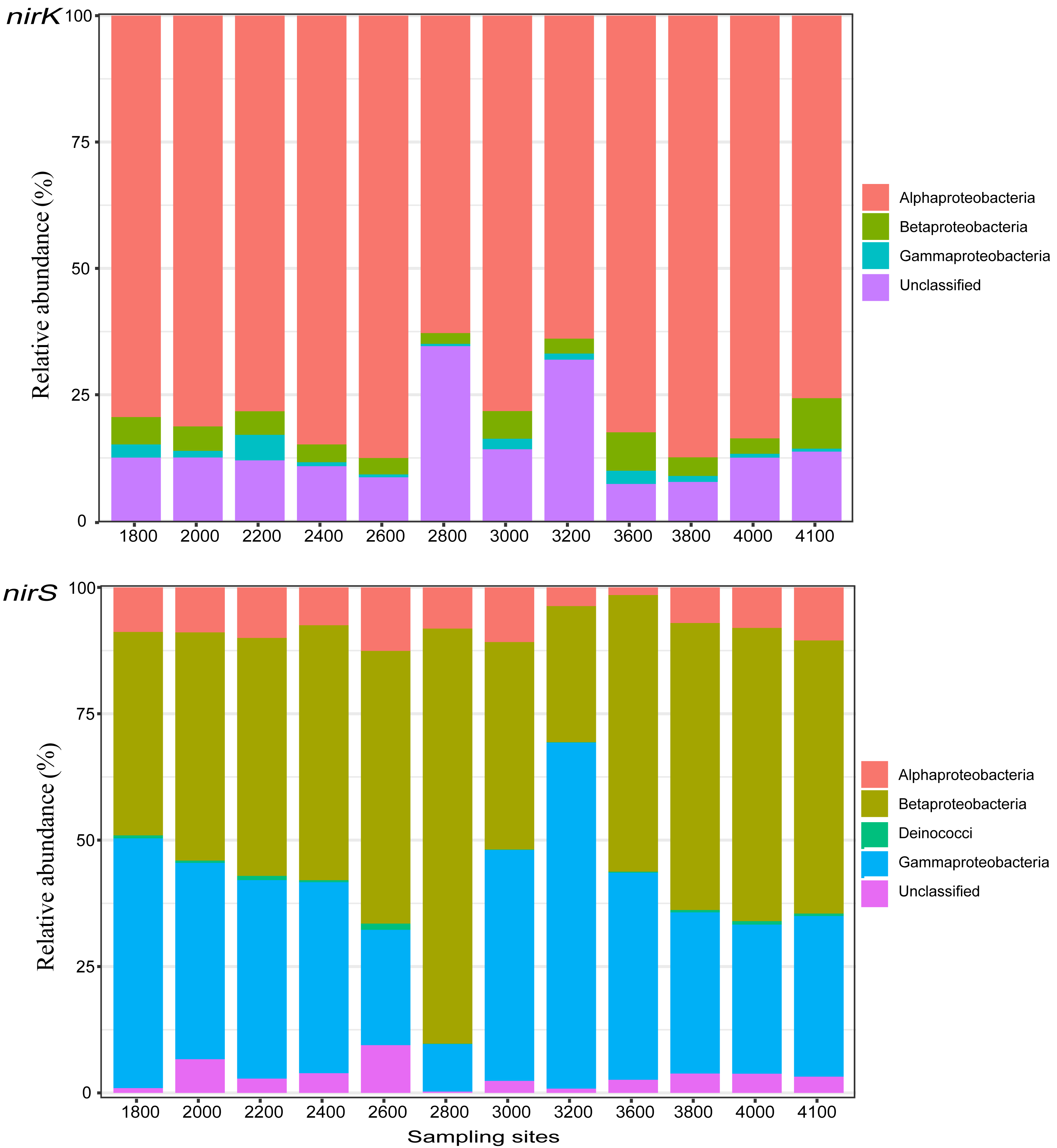

Supplement: FIG S1 [file msystems.00667-21-sf001.tif]

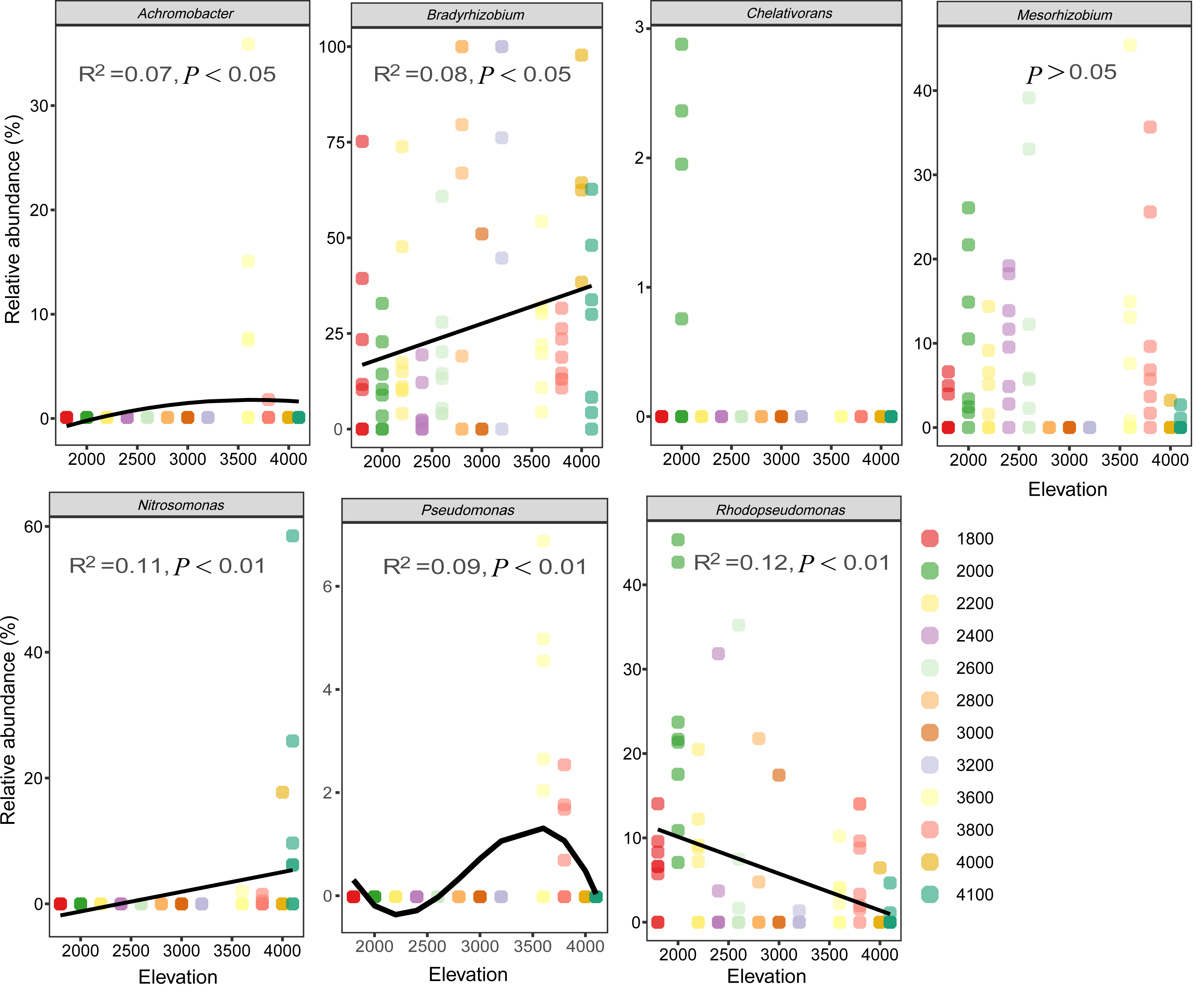

Supplement: FIG S2 [file msystems.00667-21-sf002.tif]

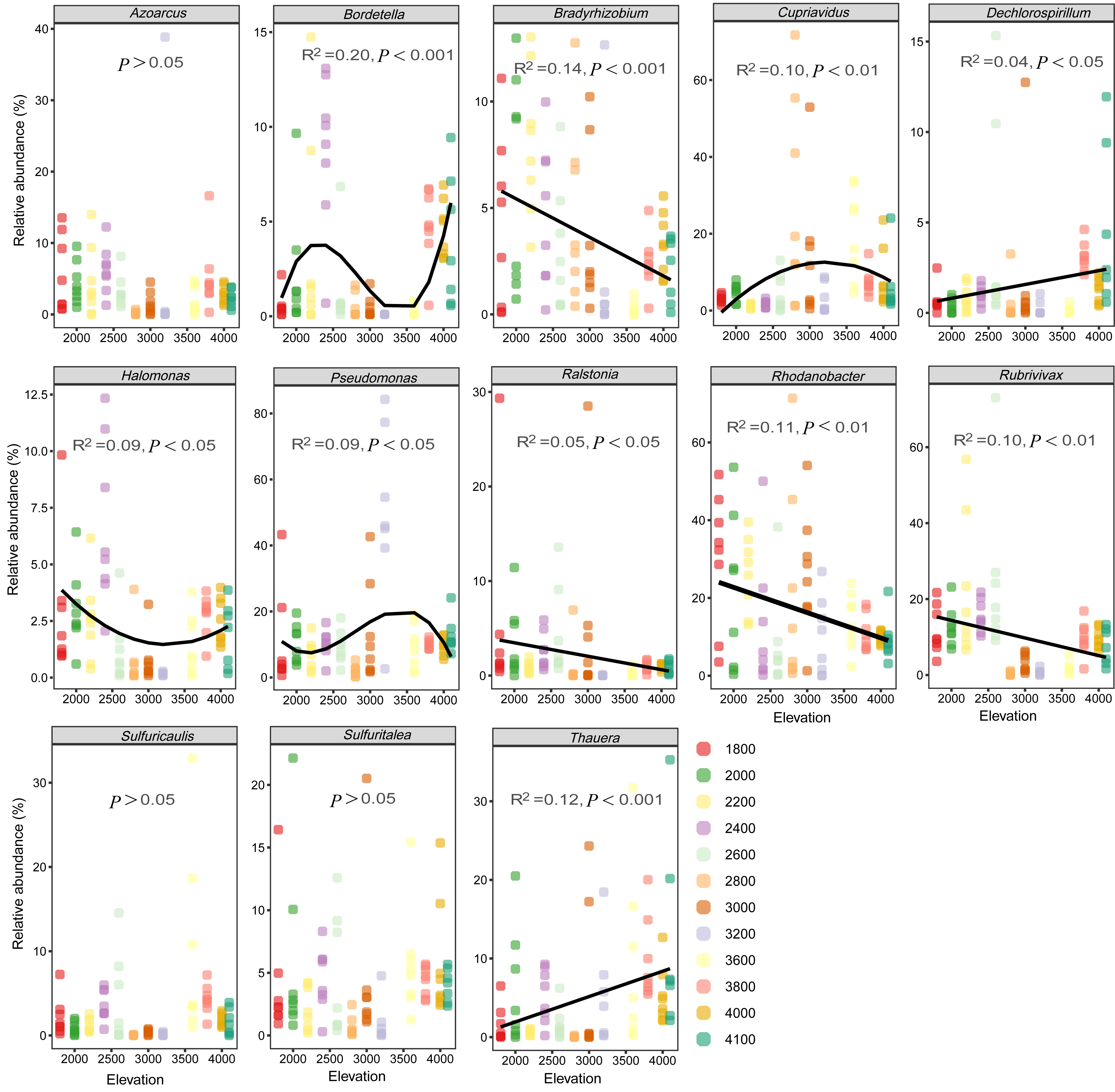

Supplement: FIG S3 [file msystems.00667-21-sf003.tif]

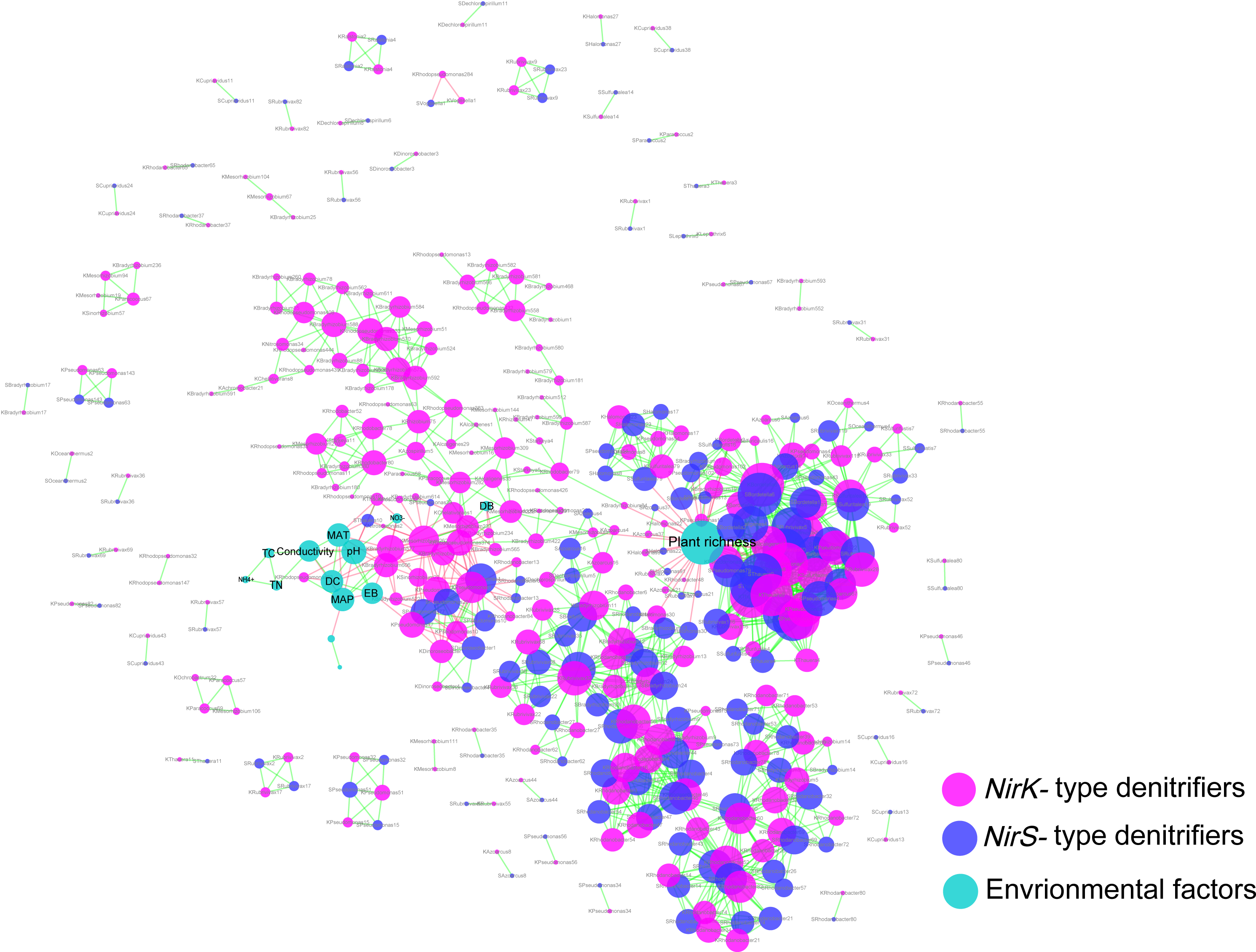

Supplement: FIG S4 [file msystems.00667-21-sf004.tif]
