# Supplementary material for: Patterns and Drivers of nirK-Type and nirS-Type Denitrifier Community Assembly along an Elevation Gradient
Source: mSystems. 2021 Nov 2;6(6):e00667-21. doi: 10.1128/mSystems.00667-21 (PMC8562487; doi:10.1128/mSystems.00667-21)
Supplement: TABLE S2 [file msystems.00667-21-st002.docx]

**TABLE S2** A non-parametric multivariate analysis of variance to test the variation in the *nirS*-type community structure (*β*- diversity) among 12 different elevations

|  | 1800 | 2000 | 2200 | 2400 | 2600 | 2800 | 3000 | 3200 | 3600 | 3800 | 4000 | 4100 |
| --- | --- | --- | --- | --- | --- | --- | --- | --- | --- | --- | --- | --- |
| 1800 | 0.00 | 1.00 | 1.00 | 0.05 | 0.77 | 0.38 | 0.82 | 0.01 | 0.01 | 0.03 | 0.02 | 0.02 |
| 2000 |  | 0.00 | 0.59 | 0.07 | 0.38 | 0.02 | 0.06 | 0.01 | 0.01 | 0.03 | 0.01 | 0.01 |
| 2200 |  |  | 0.00 | 0.10 | 0.15 | 0.03 | 0.20 | 0.01 | 0.01 | 0.01 | 0.01 | 0.02 |
| 2400 |  |  |  | 0.00 | 0.04 | 0.03 | 0.01 | 0.03 | 0.02 | 0.01 | 0.05 | 1.00 |
| 2600 |  |  |  |  | 0.00 | 0.02 | 0.02 | 0.01 | 0.02 | 0.08 | 0.01 | 1.00 |
| 2800 |  |  |  |  |  | 0.00 | 0.01 | 0.01 | 0.03 | 0.01 | 0.01 | 0.01 |
| 3000 |  |  |  |  |  |  | 0.00 | 0.12 | 0.03 | 0.07 | 0.01 | 1.00 |
| 3200 |  |  |  |  |  |  |  | 0.00 | 0.03 | 0.03 | 0.02 | 0.07 |
| 3600 |  |  |  |  |  |  |  |  | 0.00 | 0.05 | 0.02 | 0.04 |
| 3800 |  |  |  |  |  |  |  |  |  | 0.00 | 1.00 | 1.00 |
| 4000 |  |  |  |  |  |  |  |  |  |  | 0.00 | 1.00 |
| 4100 |  |  |  |  |  |  |  |  |  |  |  | 0.00 |

The data in this table represent *P* values between two elevations.
