# Supplementary material for: Patterns and Drivers of nirK-Type and nirS-Type Denitrifier Community Assembly along an Elevation Gradient
Source: mSystems. 2021 Nov 2;6(6):e00667-21. doi: 10.1128/mSystems.00667-21 (PMC8562487; doi:10.1128/mSystems.00667-21)
Supplement: TABLE S3 [file msystems.00667-21-st003.docx]

**TABLE S3** The copy numbers of *nirK* (10^8^ copies g^-1^ dry soil) and *nirS* genes (10^7^ copies g^-1^ dry soil), and the ratio of *nirK* to *nirS* genes in soil samples

| Elevation (m) | *nirK* | *nirS* | *nirK*/*nirS* |
| --- | --- | --- | --- |
| 1800 | 8.89±4.89ef | 6.61±3.15ef | 25.57±6.89 |
| 2000 | 7.66±2.72ef | 5.62±1.54ef | 24.93±8.58 |
| 2200 | 7.32±3.30de | 5.08±1.79def | 18.90±5.40 |
| 2400 | 8.70±3.05f | 6.79±1.20f | 46.72±34.08 |
| 2600 | 7.70±4.90bcde | 5.94±3.26cdef | 21.82±7.92 |
| 2800 | 5.49±4.53ef | 3.39±2.53cde | 40.29±19.68 |
| 3000 | 5.13±3.63cde | 3.80±1.94cdef | 29.16±14.80 |
| 3200 | 5.63±2.06ef | 3.53±1.92cd | 44.99±11.77 |
| 3600 | 5.44±7.49bcde | 4.11±4.70cd | 34.06±9.97 |
| 3800 | 1.40±0.46bc | 1.05±0.26a | 32.72±9.10 |
| 4000 | 3.09±1.16a | 2.00±0.45bc | 16.67±9.82 |
| 4100 | 6.06±7.19bcd | 3.08±3.89ab | 25.76±8.73 |

The values are shown as mean ± standard deviation. Values followed by different letters within the same column are significantly different at *P* < 0.05.
