# Supplementary material for: Patterns and Drivers of nirK-Type and nirS-Type Denitrifier Community Assembly along an Elevation Gradient
Source: mSystems. 2021 Nov 2;6(6):e00667-21. doi: 10.1128/mSystems.00667-21 (PMC8562487; doi:10.1128/mSystems.00667-21)
Supplement: TABLE S4 [file msystems.00667-21-st004.docx]

**TABLE S4** Spearman rank correlation analysis showing the relationships between *α*-diversity and the gene copy numbers and environmental attributes

| Environmental factors | *nirK* gene copy numbers | Observed OTUs (*nirK*) | Chao1 (*nirK*) | *nirS* gene copy numbers | Observed OTUs (*nirS*) | chao1 (*nirS*) |  |
| --- | --- | --- | --- | --- | --- | --- | --- |
|  |  |  |  |  |  |  |  |
| Longitude | 0.46** | 0.29* | 0.39** | 0.67** | 0.32** | 0.49** |  |
| Latitude | 0.49** | 0.17 | 0.29** | 0.72** | 0.33** | 0.52** |  |
| pH | 0.24* | 0.47** | 0.40** | 0.45** | 0.26* | 0.42** |  |
| NH_4_^+^N | 0.21* | -0.21 | -0.11 | 0.23* | -0.13 | -0.01 |  |
| NO_3_^-^N | 0.22* | 0.31** | 0.34** | 0.48** | 0.27* | 0.44** |  |
| TC | 0.21* | 0.12 | 0.22* | 0.39** | 0.14 | 0.28** |  |
| TN | 0.16 | 0.12 | 0.22* | 0.35** | 0.13 | 0.27* |  |
| TC/TN | 0.24* | 0.02 | 0.10 | 0.33** | 0.07 | 0.18 |  |
| Conductivity | 0.28** | 0.26* | 0.33** | 0.47** | 0.21* | 0.38** |  |
| MAT | 0.47** | 0.24* | 0.34** | 0.70** | 0.30** | 0.47** |  |
| MAP | -0.47** | -0.24* | -0.34** | -0.70** | -0.30** | -0.47** |  |
| Plant richness | 0.44** | 0.19 | 0.33** | 0.68** | 0.35** | 0.53** |  |
| DBH-DB | 0.48** | 0.21 | 0.32** | 0.71** | 0.33** | 0.52** |  |
| DBH-EB | 0.41** | 0.13 | 0.08 | 0.48** | 0.13 | 0.29** |  |
| DBH-DC | 0.28** | -0.36** | -0.23* | 0.13 | -0.25* | -0.20 |  |

TC- total carbon, TN- total nitrogen, MAT- mean annual temperature, MAP- mean annual precipitation, DBH represents the total diameter at breast height, while DBH-DB, DBH-EB and DBH-DC represent the percentage of deciduous broad trees, evergreen broad trees, and dark coniferous trees in DBH, respectively. All *P*- values of correlation analysis were adjusted using Benjamini and Hochberg false discovery rate (FDR); Significance with * *P*< 0.05, ** *P*< 0.01.
