# Supplementary material for: Patterns and Drivers of nirK-Type and nirS-Type Denitrifier Community Assembly along an Elevation Gradient
Source: mSystems. 2021 Nov 2;6(6):e00667-21. doi: 10.1128/mSystems.00667-21 (PMC8562487; doi:10.1128/mSystems.00667-21)
Supplement: TABLE S5 [file msystems.00667-21-st005.docx]

**TABLE S5** Spearman rank correlation analysis showing the relationships between the relative abundances of *nirK*-type denitrifier taxa at the genus level and environmental factors along the elevation gradient

|  | *Achromobacter* | *Bradyrhizobium* | *Mesorhizobium* | *Nitrosomonas* | *Pseudomonas* | *Rhodopseudomonas* |
| --- | --- | --- | --- | --- | --- | --- |
| Elevation | 0.18 | 0.25* | -0.06 | 0.34* | 0.09 | -0.34** |
| Longitude | -0.22 | -0.25 | 0.03 | -0.28* | -0.10 | 0.35** |
| Latitude | -0.21 | -0.23 | 0.04 | -0.30* | -0.14 | 0.33* |
| pH | -0.17 | -0.60** | 0.18 | -0.13 | -0.02 | 0.27* |
| NH_4_^+^N | 0.06 | 0.11 | -0.03 | -0.19 | -0.09 | 0.00 |
| NO_3_^-^-N | -0.13 | -0.23 | 0.01 | -0.14 | 0.02 | 0.08 |
| TC | -0.02 | -0.04 | 0.06 | -0.17 | -0.09 | 0.15 |
| TN | -0.08 | -0.07 | 0.01 | -0.16 | -0.09 | 0.18 |
| TC/TN | 0.23 | 0.01 | 0.10 | -0.17 | -0.07 | -0.04 |
| Cond | -0.07 | -0.29* | 0.10 | -0.17 | -0.11 | 0.17 |
| MAT | -0.19 | -0.24 | 0.06 | -0.34** | -0.09 | 0.35** |
| MAP | 0.19 | 0.24 | -0.06 | 0.34** | 0.09 | -0.35** |
| PR | -0.28* | -0.26 | 0.00 | -0.20 | -0.15 | 0.31* |
| DBH-DB | -0.20 | -0.30* | 0.08 | -0.23 | -0.09 | 0.23 |
| DBH-EB | 0.38** | -0.25 | 0.25 | -0.20 | 0.27* | 0.25 |
| DBH-DC | 0.05 | 0.33** | -0.18 | -0.15 | -0.05 | -0.20 |

TC- total carbon, TN- total nitrogen, Cond- Conductivity, MAT- mean annual temperature, MAP- mean annual precipitation, PR- Plant richness, DBH represents the total diameter at breast height, while DBH-DB, DBH-EB and DBH-DC represent the percentage representation of deciduous broadleaf trees, evergreen broadleaf trees and dark coniferous trees, respectively, in total DBH. All *P*- values of correlation analysis were adjusted using Benjamini and Hochberg false discovery rate (FDR); Significance with * *P*< 0.05, ** *P*< 0.01.
