# Supplementary material for: Patterns and Drivers of nirK-Type and nirS-Type Denitrifier Community Assembly along an Elevation Gradient
Source: mSystems. 2021 Nov 2;6(6):e00667-21. doi: 10.1128/mSystems.00667-21 (PMC8562487; doi:10.1128/mSystems.00667-21)
Supplement: TABLE S6 [file msystems.00667-21-st006.docx]

**TABLE S6** Spearman rank correlation analysis showing the relationships between the relative abundances of *nirS*-type denitrifier taxa at the genus level and environmental factors along the elevation gradient

|  | *Bradyrhizobium* | *Rubrivivax* | *Sulfuritalea* | *Azoarcus* | *Cupriavidus* | *Ralstonia* | *Rhodanobacter* | *Halomonas* | *Dechlorospirillum* | *Pseudomonas* | *Sulfuricaulis* | *Thauera* | *Bordetella* |
| --- | --- | --- | --- | --- | --- | --- | --- | --- | --- | --- | --- | --- | --- |
| Elevation | -0.37** | -0.31** | 0.07 | -0.14 | 0.20 | -0.23 | -0.23* | -0.21 | 0.21 | 0.10 | 0.16 | 0.33** | 0.08 |
| Longitude | 0.36** | 0.34** | -0.06 | 0.13 | -0.20 | 0.22 | 0.22* | 0.23 | -0.17 | -0.16 | -0.17 | -0.32** | -0.04 |
| Latitude | 0.38** | 0.36** | -0.07 | 0.12 | -0.22 | 0.21 | 0.23* | 0.19 | -0.18 | -0.11 | -0.17 | -0.34** | -0.06 |
| pH | 0.11 | 0.46** | 0.03 | 0.32** | -0.43** | 0.08 | -0.03 | 0.51** | 0.07 | 0.01 | -0.02 | 0.14 | 0.36** |
| NH_4_^+^-N | 0.04 | 0.06 | -0.25* | -0.02 | 0.09 | 0.19 | -0.03 | -0.28* | 0.06 | 0.07 | 0.01 | -0.07 | -0.25* |
| NO_3_^-^-N | 0.18 | 0.45** | -0.04 | 0.17 | -0.28* | 0.08 | 0.11 | 0.11 | -0.12 | -0.06 | -0.01 | -0.24* | 0.05 |
| TC | 0.15 | 0.49** | -0.20 | 0.03 | -0.08 | 0.13 | -0.02 | -0.03 | -0.01 | -0.19 | 0.02 | -0.28* | 0.01 |
| TN | 0.20 | 0.47** | -0.23 | 0.06 | -0.14 | 0.16 | 0.02 | -0.04 | -0.01 | -0.17 | -0.07 | -0.29* | 0.03 |
| TC/TN | -0.04 | 0.19 | 0.04 | 0.04 | 0.12 | 0.04 | -0.13 | 0.28* | -0.11 | -0.06 | 0.16 | -0.14 | 0.10 |
| Cond | 0.11 | 0.52** | -0.14 | 0.18 | -0.24* | 0.15 | -0.11 | 0.23 | 0.00 | -0.11 | -0.02 | -0.15 | 0.28* |
| MAT | 0.38** | 0.31** | -0.08 | 0.13 | -0.19 | 0.23 | 0.23* | 0.17 | -0.21 | -0.11 | -0.16 | -0.34** | -0.09 |
| MAP | -0.38** | -0.31** | 0.08 | -0.13 | 0.19 | -0.23 | -0.24* | -0.17 | 0.21 | 0.11 | 0.16 | 0.34** | 0.09 |
| PR | 0.40** | 0.39** | -0.05 | 0.14 | -0.28* | 0.24* | 0.20 | 0.28* | -0.12 | -0.17 | -0.22 | -0.32** | 0.04 |
| DBH-DB | 0.37** | 0.50** | -0.04 | 0.17 | -0.32** | 0.18 | 0.21 | 0.30** | -0.09 | -0.17 | -0.08 | -0.32** | 0.09 |
| DBH-EB | -0.02 | 0.00 | 0.15 | 0.07 | 0.05 | -0.01 | 0.08 | 0.23 | -0.29* | -0.05 | 0.34** | 0.09 | 0.01 |
| DBH-DC | -0.11 | -0.38** | -0.20 | -0.16 | 0.30** | 0.02 | -0.07 | -0.46** | -0.08 | 0.25* | -0.14 | -0.10 | -0.44** |

TC- total carbon, TN- total nitrogen, Cond- Conductivity, MAT- mean annual air temperature, MAP- mean annual precipitation, PR- Plant richness, DBH represents the total diameter at breast height, while DBH-DB, DBH-EB and DBH-DC represent the percentage representation of deciduous broadleaf trees, evergreen broadleaf trees and dark coniferous trees, respectively, in total DBH. All *P*- values of correlation analysis were adjusted using Benjamini and Hochberg false discovery rate (FDR); Significance with * *P*< 0.05, ** *P*< 0.01.
